# Make Home Happy

**Published:** 1857-02

**Authors:** 


### MAKE HOME HAPPY

Parents, if you wish to prevent your children from falling into practices and associations which lead to loss of health and morals, and to a premature grave. The love of home, as a part of parental teaching, forms the subject of an article in that very excellent publication, *The Presbyterian Magazine*, of Philadelphia; and we trust that all who read it will give it adequate consideration. It is not enough that our children have abundant food and clothing, and comfortable lodging. There is a monotony about these things which soon tires; the very absence of such comforts is an agreeable relief at any time if away from home. It is a common remark, that a child eats almost as much as a grown person, and nothing will satisfy a hungry child. It is strikingly so with the mind; it must have food to feed it; that food is variety; the variety of the new, the unknown; that is what delights children of all ages; and to gratify that delight by presenting to their attention, with *moderate rapidity* of succession, what is substantial, valuable, practical, is one of the most important of all parental occupations. And parents should feel themselves constantly stimulated to efforts of this kind by the consideration, that if they do not hold these things up to their attention, their reverses will be presented to them in endless combinations, by the lower associations of the street and of the kitchen.

The three necessities of children are food, exercise, amusement. They will eat, they will move about, they will be entertained. The feeding of the mind is as essential as the feeding of the body, and not half a parent's duty is done in securing house, and food, and raiment. So far from appreciating this mental necessity, we are too apt to thwart their own instinctive efforts to satisfy it, by our short and listless, if not, indeed, impatient and angry answers to their multitudinous inquiries. Under such treatment, they soon learn the uselessness of seeking information from their parents, and gradually seek it elsewhere, with its large admixture of incorrectness, imperfectness, and, too often, viciousness.

In our opinion, neither sons nor daughters should be allowed to sleep away from home, unless their parents are with them. We sincerely hope that such a blessing may be secured to ours, until the day of marriage. It is a true mother's love which seeks to keep her daughter in sight until superior claims come; it would save many a family from social ruin, and many a parent's heart from breaking. As for our sons, it should be impressed upon them that no business is to require their attention and to keep them out of the house after sundown, unless the parent is along, as long in their teens as it is possible to secure obedience to such a requisition. And to make such obedience pleasurable, let it be the parents' study to render home inviting by the cultivation of all that is courteous and kindly, and by the large and habitual exercise of the better qualities of our nature, especially those of sympathy, and love, and affection. To all parents we say—

Keep your children at home as much, and together, as long as it is at all possible for you to do it. No better plan can be devised for enabling a household to grow up loving and being loved, in all its members.

